# Camel (*Camelus dromedarius* L. and *Camelus bactrianus* L.) Milk Composition and Effects on Human Type 1 and Type 2 Diabetes Mellitus: A Review

**DOI:** 10.3390/biology14091162

**Published:** 2025-09-01

**Authors:** Massimo Faustini, Daniele Vigo, Gabriele Brecchia, Stella Agradi, Susanna Draghi, Giulio Curone, Moufida Atigui, Amel Sboui, Alda Quattrone, Nour Elhouda Fehri

**Affiliations:** 1 Department of Veterinary Medicine and Animal Sciences, University of Milan, Via dell’Università 6, 26900 Lodi, Italy; massimo.faustini@unimi.it (M.F.); daniele.vigo@unimi.it (D.V.); gabriele.brecchia@unimi.it (G.B.); stella.agradi@unimi.it (S.A.); susanna.draghi@unimi.it (S.D.); giulio.curone@unimi.it (G.C.); nour.fehri@unimi.it (N.E.F.); 2 Livestock and Wildlife Laboratory, Arid Regions Institute, IRESA, Medenine 4100, Tunisia; atigui2009@gmail.com (M.A.); amelsb8@gmail.com (A.S.)

**Keywords:** camel milk, diabetes mellitus, therapeutic properties, nutritional value

## Abstract

This review highlights the anti-hyperglycemic and antidiabetic properties of camel and dromedary milk (CM). Diabetes mellitus poses a significant global health challenge, and strategies that reduce reliance on insulin or other medications could substantially improve patient management. CM could represent a promising complementary approach due to its established antidiabetic effects, which are supported by its unique biological characteristics. Compared to other common milks, such as bovine milk, CM contains higher concentrations of insulin. Its distinctive physicochemical and microstructural properties help protect insulin and other bioactive proteins from degradation in the gastrointestinal tract, thereby enhancing their intestinal absorption. Furthermore, peptides generated during CM protein digestion may exert direct or indirect effects on the liver and pancreas, contributing to improved glucose metabolism. These beneficial actions are further supported by CM’s antioxidant and antilipidemic properties, which may help mitigate diabetes-related complications, including renal dysfunction and skin lesions.

## Simple Summary

Camel milk has gained attention for its potential role in diabetes management due to its unique proteins and bioactive compounds that may help regulate blood glucose levels. Clinical studies in both type 1 and type 2 diabetes patients suggest that regular consumption of camel milk can lower fasting glucose, enhance insulin activity, and potentially decrease the need for exogenous insulin or other diabetes medications. Moreover, its antioxidant and anti-inflammatory properties may support overall metabolic health and help mitigate diabetes-related complications. As a natural product, camel milk shows promise as a complementary therapy alongside conventional diabetes treatments. Nevertheless, further large-scale, well-designed clinical trials are needed to confirm their efficacy and elucidate the mechanisms underlying their beneficial effects. Overall, camel milk may offer meaningful advantages for glycemic control and diabetes management and warrants additional research for broader applications in other metabolic conditions.

## 1. Introduction

Diabetes mellitus (DM) is a major global public health issue, with an estimated 589 million adults affected worldwide in 2024, a number expected to reach 783 million by 2045, and 853 million by 2050 among individuals aged 20–79 years [1]. Type 2 diabetes mellitus (T2DM) accounts for approximately 90% of all diabetes cases and is strongly linked to lifestyle factors such as sedentary behavior, unhealthy diets, and obesity [2]. The remaining cases include mainly Type 1 diabetes mellitus (T1DM), latent autoimmune diabetes in adults (LADA), and maturity-onset diabetes of the young (MODY) [3].

Diabetes is associated with a wide range of complications affecting multiple organ systems, including microvascular damage such as retinopathy, nephropathy, and neuropathy, as well as macrovascular events like cardiovascular disease and stroke. Additionally, diabetes impairs skin integrity and delays wound healing, further contributing to morbidity [3]. These complications contribute to significant disability, decreased quality of life, and increased economic burden on healthcare systems. In recent years, there has been growing interest in the role of functional foods as adjunctive strategies for diabetes management, alongside conventional therapies such as insulin administration, oral hypoglycemic agents, and lifestyle modifications [4]. Among these functional foods, camel and dromedary milk (CM) has attracted attention for its potential antidiabetic properties. CM is naturally rich in insulin-like proteins, bioactive peptides, antioxidants, and immunomodulatory compounds [5]. Its unique physicochemical and microstructural characteristics facilitate the protection and intestinal absorption of these bioactive molecules, potentially enhancing their systemic effects [6]. Although the underlying mechanisms are not yet fully elucidated, current evidence suggests that CM can improve pancreatic β-cell function, improve insulin sensitivity, modulate hepatic glucose metabolism, and reduce oxidative stress [7]. This wide range of bioactivities positions CM as a potential adjunct in diabetes management and as a candidate for the prevention or treatment of other metabolic and inflammatory disorders. According to Liu et al. [8], its key functional properties include:

- Inhibition of angiotensin-converting enzyme (ACE), indicating a potential role in blood pressure regulation.
- Antiproliferative activity against immortalized cancer cell lines, supporting its potential anticancer effects.
- Amelioration of metabolic symptoms associated with diabetes, including the following:
  - Reduction in fasting blood glucose levels;
  - Decreased insulin resistance, as measured by the Homeostasis Model Assessment of Insulin Resistance (HOMA-IR);
  - Improvement of blood lipid profiles, notably through the reduction of triglycerides and cholesterol;
  - Decreased plasma levels of LDL-C (Low-Density Lipoprotein Cholesterol) and VLDL-C (Very-Low-Density Lipoprotein Cholesterol), along with an increase in HDL-C (High-Density Lipoprotein Cholesterol) concentrations.
- Anticoagulant and antithrombotic properties, evidenced by the following:
  - Reduction in coagulopathy manifestations;
  - Improved platelet function.
- Antioxidant capacity, as indicated by the reduction in oxidative stress markers;
- Regenerative effects on pancreatic β-cells, potentially contributing to glycemic control in diabetic patients;
- Hepatoprotective and nephroprotective activities, including the attenuation of liver injuries and steatohepatitis, as well as protection against renal damage.

These findings highlight the multifunctional bioactivity of CM, supporting its potential as a functional food with therapeutic applications across metabolic, cardiovascular, hepatic, renal, and oncological health contexts.

This review summarizes current knowledge on the composition and bioactive properties of CM, with particular emphasis on its potential as an adjunct to pharmacological therapy in the management of DM. For a comprehensive and up-to-date review of the therapeutic functions of CM, see Sharma et al. [9].

## 2. Materials and Methods

### Bibliographic Search Description

A comprehensive literature search was conducted across major databases, including Scopus, Web of Science, and Google Scholar, covering publications up to July 2025. The search strategy combined the following keywords: (“camel” OR “dromedary”) AND “milk” AND “diabetes”, and (“camel” OR “dromedary”) AND “milk” AND (“glycemia” OR “glycaemia”), and (“camel milk”) AND (“diabetes mellitus”).

Following the initial search, articles were screened for relevance based on their titles and abstracts. Inclusion criteria were: (i) original research, review articles, or meta-analyses published in peer-reviewed journals; (ii) studies evaluating camel or dromedary milk in relation to diabetes or glucose metabolism; (iii) publications in English; and (iv) studies involving human subjects, animal models, or in vitro experiments. Publications detailing the composition of camel milk or its therapeutic effects on diabetes were also considered.

After this screening, potentially relevant articles were retrieved in full text for detailed evaluation. Duplicate publications were removed, resulting in a final selection of 166 relevant articles. These studies were subsequently classified into the following thematic categories: (1) compositional and functional characteristics of camel and dromedary milk; (2) general aspects of diabetes mellitus, including its pathophysiology, classification, diagnostic criteria, epidemiology, and conventional management; (3) effects of camel/dromedary milk on Type 1 and Type 2 diabetes; and (4) proposed mechanisms of action underlying the antidiabetic properties of camel/dromedary milk. Illustrations were created using the online scientific tool BioRender (www.biorender.com, accessed on 12 July 2025).

## 3. Camel Milk Production

Camel and dromedary milk (CM) has long been a staple food in arid and semi-arid regions of the Middle East, North Africa, and parts of Asia. Its consumption is now expanding due to increasing recognition of its potential health benefits, particularly in the context of metabolic and autoimmune diseases [10].

Camels belong to the genus *Camelus*, which includes two domesticated species: the one-humped dromedary (*Camelus dromedarius* L.) and the two-humped Bactrian camel (*Camelus bactrianus* L.). These species encompass numerous breeds distributed across diverse geographical areas of Africa, the Middle East, Central Asia, and China. The significant genetic diversity among these breeds, combined with environmental, physiological, and management factors, results in substantial variation in milk production traits [10,11]. Camel lactation typically lasts between 6 and 18 months, with an average duration of approximately 12 months, following a gestation period of 390 days (13 months). Gestation length can be influenced by factors such as fetal sex and weight, season of parturition, and species, with Bactrian camels generally having longer gestations than dromedaries [12].

Globally, camels represent only 0.4% of the global domestic herbivore population [13], yet their numbers are steadily increasing. FAO data from 1961 to 2022 indicate an average annual growth rate of 6.5% in camel milk production. In 2022, global production reached 4,116,669 tons, reflecting a 0.83% annual increase from 3,430,675 tons in 2014. According to FAOSTAT (2022), Kenya is the largest producer of raw camel milk, followed by Somalia, Pakistan, Mali, Ethiopia, Saudi Arabia, Niger, and the United Arab Emirates (Table 1) [14].

Accurately estimating average milk yield in camels is particularly challenging due to significant differences in management systems, feeding regimens, environmental conditions, and milk collection methodologies. A significant portion of available data originates from experimental stations, leaving yields under pastoral and extensive systems largely undocumented [15]. Furthermore, inconsistencies in milk recording protocols, such as the frequency of milking and the proportion of milk reserved for the calf, make comparisons between different camel breeds and production systems even more complex [11,15]. Nonetheless, available data suggest that Asian dromedary breeds generally exhibit a higher milk yield potential compared to their African counterparts, which tend to produce less under similar conditions. For instance, in the United Arab Emirates, milk production in intensively managed camels was estimated at 3314 ± 98.5 L over an average lactation period of 586 ± 11.0 days [16]. In contrast, African camels typically yield between 1000 and 2700 L per lactation under more extensive or semi-intensive systems [11]. In Egypt, the Maghrebi camel has been reported to produce an average of 1612 ± 710 L during a lactation period of 353 ± 152 days [17], while Tunisian Maghrebi camels recorded a higher average yield of 2642 ± 523 L over a lactation duration of 390 days [18]. In Ethiopia, a small observational study (*n* = 5) estimated an annual yield of 1123 L per camel under the traditional management system [19]. These findings are summarized in Table 2. As previously noted, these numbers are estimates that reflect the challenges in systematic data collection.

To standardize milk yield comparisons, Zhang et al. [20] proposed a classification system based on annual production: high producers (>3000 L/year), medium producers (1500–3000 L/year), and low producers (<1500 L/year). This framework effectively captures the vast spectrum of productivity observed globally. High-producing examples include certain Pakistani breeds, with reported yields of 2440–10,675 L over 305 days, with lactations lasting between 12 and 35 months, and daily production of 8 to 20 L under optimal conditions [21]. At the opposite end, Mongolian camels have been reported to produce approximately 477 L per 305 days, with lactation periods extending up to 16 months, and daily yields of only 1–2 kg [10]. These pronounced disparities reflect the combined influence of genetics, management practices, and environmental conditions on lactation performance, reinforcing the challenge of making direct comparisons across different camel populations and production systems.

**Table 2:** Camel milk yield and lactation duration across different regions.

Region/Country	Milk Yield (Liters per Lactation)	Lactation Duration	No. of Animals	Ref.
Mongolia	477	Up to 16 months	-	[10]
Africa	1000–2700	-	-	[11]
Egypt (Maghreb)	1612 ± 710	353 ± 152 d	43 (748 records)	[17]
Tunisia (Maghreb)	2642 ± 523	390 d	26 lactations	[18]
Ethiopia	1123	-	5	[19]
Pakistan	2440–10,675	12–35 months	-	[21]

## 4. Camel Milk’s Gross Composition

Milk is a fundamental component of human nutrition and is widely consumed worldwide. While bovine milk remains the most extensively consumed due to its high availability and economic significance, global interest in alternative milk sources has increased considerably in recent years [22]. This trend is driven not only by cultural and regional dietary habits but also by growing awareness of the unique nutritional and functional properties of non-bovine milks, including camel milk [22].

Until a few decades ago, the scientific literature on CM was relatively limited, with early investigations focusing primarily on its chemical composition [23,24] and production systems [11]. More recent studies have explored its potential health benefits, notably its hypoallergenic properties [25], as well as reported anticancer [26] and antidiabetic activities [27]. These functional effects are thought to arise from the presence and biological activity of various macromolecules, such as proteins, lipids, and polysaccharides, as well as their digested products. From a nutritional perspective, some of CM’s beneficial properties have been linked to its relatively high proportion of unsaturated fatty acids [28].

CM is also characterized by distinctive protein features, including a low content of κ-casein and the absence of β-lactoglobulin in its whey fraction, which may contribute to its reduced allergenicity [29]. Furthermore, the presence of bioactive components such as immunoglobulins, lactoferrin, and vitamin C enhances its nutritional and therapeutic value [30,31], supporting its potential role in both health promotion and disease management.

In terms of gross composition, Gnan and Sheriha [32] reported substantial regional variability, with protein content ranging from 3.5 to 4.5%, lactose from 3.4 to 5.6%, fat from 3.07 to 5.50%, ash from 0.7 to 0.95%, and total solids between 12.1 and 15.0%.

A meta-analysis by Konuspayeva et al. [33], synthesizing 82 bibliographic sources on the milk yield of Bactrian, dromedary, and hybrid camels from different regions, provides an overview of CM chemical components. The descriptive statistics from their review, further analyzed in the present work, are presented in Table 3, with selected findings discussed in the main text.

Recently, Benmeziane-Derradji [14] reviewed the compositional characteristics of CM using bibliographic data from 21 sites across 10 countries in the Middle East and North Africa (MENA) region. The reported mean ± standard deviation (SD) values were as follows: protein 3.03 ± 0.56% (range: 2.15–4.61%), fat 3.27 ± 0.55% (range: 2.31–4.28%), lactose 4.26 ± 0.61% (range: 2.93–5.12%), ash 0.80 ± 0.17% (range: 0.60–1.30%), and water 88.18 ± 1.84% (range: 83.5–90.66%). Water content, averaging approximately 88%, was strongly influenced by variations in the animals’ water intake [14].

### 4.1. Protein

The protein content of CM shows considerable variability, ranging from 2.15% to 4.61%, with an overall mean of approximately 3.03%, making it an important source of high-quality protein for populations in arid and semi-arid regions [14]. Regional differences are evident: in the United Arab Emirates, protein levels range from 3.5% to 4.5%, with a mean of 3.71% [34]; in Egypt, from 3.0% to 4.5% with a mean of 4.02% [35]; in Tunisia, from 3.16% to 4.37% [36]; and in Morocco, from 3.22% to 3.50% [37].

Caseins represent the dominant protein fraction in CM, accounting for 52% to 87% of total proteins [38]. The casein profile comprises αs1-, αs2-, and β-caseins, while the κ-casein fraction is notably low compared to bovine milk [39,40]. Although five κ-casein isoforms have been identified in CM [41], they represent only about 5% of total caseins, significantly less than the approximately 13% κ-casein content found in bovine milk [42].

A key distinctive feature of CM is the absence of β-lactoglobulin, a major whey protein that constitutes about 50% of the whey fraction in bovine milk [43,44,45]. This absence significantly contributes to CM’s hypoallergenic properties, making it a suitable alternative to bovine milk for individuals with cow milk protein allergies [25].

### 4.2. Fat

The fat content of CM, as in other ruminants, is influenced by multiple factors including breed, physiological status, season, diet, reproductive condition, and lactation stage [46]. Benmeziane-Derradji [14] reported an overall mean fat content of 3.27%, although seasonal variations have been observed, with Saudi CM showing lower fat levels (2.29%) during the hot season and higher values (3.46%) in January [47]. In dromedary camels, fat content varies widely, ranging from 2.56% to 5.80% [46,48].

The fatty acid (FA) profile of CM is characterized predominantly by saturated fatty acids, mainly myristic and palmitic acids, along with odd-numbered, unsaturated long-chain and short-chain FAs. In particular, saturated FAs constitute 46.41% to 65.08% of total fat, monounsaturated FAs 28.18% to 49.31%, and polyunsaturated FAs (PUFAs) 1.97% to 4.26% [14]. Notably the overall proportion of unsaturated FAs in CM exceeds that of bovine milk (53.9% vs. 39.24%, respectively) [49], consistent with findings by Konuspayeva et al. [50], who reported 33.6% unsaturated FA in camel milk compared to 24.1% in cow’s milk. Additionally, saturated FA content is correspondingly lower in CM, contributing to its favorable nutritional profile [46].

Particular nutritional interest in CM also stems from its relatively high PUFA content, which is essential for human health. Ayadi et al. [51] reported total unsaturated FA content of approximately 54%, with PUFAs constituting about 5%. Within this, n-6 fatty acids accounted for 4.3%, n-3 for 0.7%, resulting in an n-6/n-3 ratio of 7. Their study also highlighted the significant influence of both the breeding system and lactation stage on the FA profile. Furthermore, Chamekh et al. [52] showed that lactation stage impacts the profile of odd- and branched-chain fatty acids, with mid-lactation profiles differing notably from early and late lactation stages. These authors also found that camels reared under semi-intensive systems exhibited a more favorable FA profile compared to those managed intensively [52].

### 4.3. Lactose

Lactose, the main carbohydrate in ruminant milk, exhibits considerable variability, ranging from 2.93% to 5.12%, with an average value of approximately 4.26% [14]. A study on Algerian camels reported a lactose content of 4.31%, which is slightly lower than the typical 5.05% found in cow milk [53]. Similarly, Egyptian CM has been reported to contain about 4.86% lactose [54]. However, lactose concentrations can sometimes reach notably low levels; for instance, Shuiep et al. [55] found lactose contents of 3.12% and 2.90% in camels from Eastern Nile and Western Omdurman regions of Sudan, respectively. In contrast, Yoganandi et al. [56] observed unusually high lactose percentages, 7.56% and 7.04%, in CM from Indian districts.

As with other milk components, lactose content is influenced by multiple factors, including seasonal variations and the mobility status of the animals, reflecting environmental and physiological adaptations [55,57].

### 4.4. Vitamins

CM contains measurable amounts of vitamins A, D, and E; however, one of its most distinctive nutritional characteristics is its exceptionally high vitamin C content [58]. The concentration of ascorbic acid in CM is reported to be approximately 30 times higher than in cow milk and about six times higher than in human milk, making it a vital dietary source of vitamin C for populations living in arid regions where access to fresh fruits and vegetables is limited [58,59]. Farah et al. [60] estimated the average vitamin C content in CM at 37.4 mg/L.

### 4.5. Minerals

As in other mammalian species, CM is rich in minerals, particularly calcium and phosphorus, with total mineral content ranging from 0.60% to 1.30% and a mean value of 0.80% [14]. Significant regional and seasonal variations have been reported. For instance, Konuspayeva et al. [61] reported a mean calcium concentration of 1.232 g/L, whereas Mostafidi et al. [62] found higher calcium levels in camels reared in desert conditions compared to more favorable feeding environments. The calcium content in Bactrian CM appeared significantly higher (1.30 ± 0.29 g/L) compared to dromedary milk (1.16 ± 0.27 g/L), while crossbreed Bactrian*dromedary showed an intermediate value (1.23 ± 0.27 g/L) [33].

Compared to sheep and goat milk, CM generally has lower calcium content but higher levels of sodium, potassium, and copper [63,64]. In a comprehensive study, Alhaj et al. [65] reported the composition of CM calculated by a meta-analytic and meta-regressive statistical process, considering breed, season, year, and country as influencing factors. The mean composition in Bactrian and dromedary camels, derived from the analysis of 79 selected bibliographic sources is reported in Table 4. The authors concluded that observed differences among studies reflect variations in breed, location, season, sample size, management, feeding, and water availability [65].

### 4.6. CM Composition Variation During Lactation

As in other mammalian species, CM undergoes substantial compositional changes throughout lactation. Zhang et al. [20] reported pronounced alterations in the mammary secretions of Alxa camels during the first week postpartum. Protein concentration declined sharply within the first 12 h after parturition, from 14.23% to 9.63%, reaching 7.17% by the second day, and stabilizing at approximately 3.55% by day 90 of lactation. In contrast, lactose levels remained relatively stable during the first three months, ranging from 4.24% to 4.44% [20].

Fatty acid composition also showed notable changes over lactation. For instance, Bactrian camel colostrum was characterized by a predominance of even-numbered saturated fatty acids and higher polyunsaturated fatty acid content compared to mature milk [20]. Mineral concentrations exhibited dynamic fluctuations as well. Calcium levels decreased markedly within the first 24 h after delivery, followed by a slight increase by day 7 and a gradual decline to 154.57 mg/dL by day 90. Phosphorus followed a similar trend, although its concentrations were consistently lower than those of calcium [20].

Across various studies, the concentrations of major minerals and electrolytes in dromedary milk, such as calcium, phosphorus, sodium, and potassium, ranged from 23 to 214 mg/100 g [23,48,58,64], often exceeding those reported in bovine milk, particularly around day 90 postpartum [20].

Dromedary colostrum also exhibited dynamic changes during early lactation. Konuspayeva et al. [66] reported higher levels of basic components, such as fat and proteins, compared to overall averages from their earlier meta-analysis [33]. In the immediate postpartum period, there was a notable decrease in dry matter, density, and protein content within the first seven days. Specifically, total fat dropped from 25.9% to 3.1%, while total protein declined from 17.2% to 4.2% [66], as summarized in Table 5.

#### Camel Milk: A Putative Functional Food in Supporting Diabetes Mellitus?

Milk represents a highly complex biological system, comprising diverse molecules whose functional effects depend not only on their individual concentrations but also on their interactions. CM, due to its distinctive chemical and biochemical composition, has been proposed as a dietary adjuvant in the management of all forms of diabetes mellitus. Its composition differs substantially from that of cow and sheep milk, which may contribute to the lower prevalence of diabetes observed in populations where CM is regularly consumed, either as fresh milk, fermented products, or other derivatives [67].

In particular, CM exhibits unique physicochemical and bioactive characteristics. It does not coagulate readily under acidic conditions, a trait potentially linked to its low degree of casein phosphorylation [54]. CM is also low in cholesterol and contains a relatively high proportion of polyunsaturated fatty acids in a naturally emulsified form, enhancing their bioavailability [67]. Additionally, CM has reduced κ-casein content and completely lacks β-lactoglobulin, proteins commonly implicated in allergenic reactions to bovine milk, thereby lowering the risk of milk-induced allergies [68,69]. These features have fueled growing scientific interest in CM as a functional food with potential benefits for glycemic regulation and overall metabolic health.

## 5. Diabetes Mellitus in Humans: Classification and Prevalence

Diabetes mellitus is a group of disorders characterized by chronic hyperglycemia resulting from impaired insulin secretion, insulin action, or a combination of both [3]. The clinical presentation and progression vary considerably, reflecting the complex and multifactorial nature of the disease. According to the American Diabetes Association (ADA) Standards of Care in Diabetes—2025 [3], diabetes in humans is classified into four major forms:

- *Type 1 Diabetes Mellitus* (T1DM): caused by autoimmune destruction of pancreatic β-cells, usually leading to absolute insulin deficiency. This category includes latent autoimmune diabetes in adults (LADA);
- *Type 2 Diabetes Mellitus* (T2DM): the most prevalent form of diabetes, characterized by insulin resistance combined with a relative and progressively worsening β-cell dysfunction. It is strongly associated with obesity, sedentary lifestyle, and genetic predisposition and often occurs in the context of metabolic syndrome;
- *Specific types of diabetes due to other causes*: these include monogenic forms of diabetes (e.g., neonatal diabetes, maturity-onset diabetes of the young [MODY]), diseases affecting the exocrine pancreas (e.g., cystic fibrosis, chronic pancreatitis), and diabetes secondary to medications or chemicals (e.g., prolonged glucocorticoid therapy, antiretroviral treatment, or immunosuppressive agents used in organ transplantation);
- *Gestational diabetes mellitus* (GDM): a form of hyperglycemia manifested during the second or third trimester of pregnancy, in individuals without previously diagnosed diabetes. It affects an estimated 21.1 million pregnancies worldwide, increasing the risk of complications for both mother and fetus, and elevating the mother’s future risk of developing T2DM.

In humans, normal reference values for blood glucose range between 70 mg/dL (3.9 mmol/L) and 100 mg/dL (5.6 mmol/L) (World Health Organization, WHO website [70]). The principal diagnostic criteria for DM include a fasting plasma glucose level ≥126 mg/dL (7.0 mmol/L) or a glycated hemoglobin (HbA1c) value ≥6.5% (48 mmol/mol). Additional diagnostic criteria include a blood glucose concentration ≥200 mg/dL (11.1 mmol/L) two hours after the oral administration of 75 g of anhydrous glucose during an oral glucose tolerance test (OGTT), or a random plasma glucose level ≥200 mg/dL (11.1 mmol/L) in the presence of symptoms of hyperglycemia [3].

Chronic hyperglycemia, the hallmark of diabetes, is associated with a broad spectrum of complications involving the kidneys, cardiovascular system, eyes, nervous system, and skin. Effective glycemic control, achieved through the administration of insulin, hypoglycemic agents, appropriate dietary management, and physical activity, is essential to reduce the risk of these long-term complications and improve patient outcomes [71].

As of 2024, an estimated 589 million adults aged 20–79 years worldwide (approximately 11.1% of the global population in that age group) are living with DM [1]. This represents a significant increase from 2021, when diabetes affected approximately 536.6 million people, with a global prevalence of 10.5% [72]. Among adults in 2024, an estimated 252 million (43%) remained undiagnosed, highlighting a substantial gap in detection and care. Additionally, 635 million adults (12%) exhibited impaired glucose tolerance (IGT), and 488 million (9.2%) had impaired fasting glucose, both of which are strong risk factors for future diabetes onset. The pediatric population is also affected, with approximately 1.81 million children and adolescents (aged 0–19 years) currently living with type 1 diabetes, a notable increase from the 1.2 million cases reported in 2021. Projections by the International Diabetes Federation suggest that the total number of adults with diabetes will rise to 643 million by 2030 and exceed 853 million (13.0%) by 2050, with more than 80% of affected individuals residing in low- and middle-income countries [1]. This growing prevalence underscores the urgent need to identify and develop therapeutic adjuvants to support glycemic control and improve disease management.

## 6. Effects of Camel Milk on Diabetes Mellitus

One of the most compelling aspects of CM research lies in the diverse bioactive molecules and their derivatives that may contribute to its health-promoting effects in both animals and humans [73]. Ongoing studies aim to determine whether these effects are mediated by intact native bioactive compounds, by digestion-derived peptides, or by a combination of both. The bioactivity of these molecules is influenced by their intrinsic structural properties as well as by the physiological conditions of the gastrointestinal environment. Notably, CM appears capable of protecting certain proteins from degradation in the gastric and intestinal tract, thereby facilitating their absorption through the intestinal barrier. Consequently, the beneficial actions of CM may result from the synergistic activity of multiple molecular components acting in concert. In the context of DM, the therapeutic effects of CM are primarily exerted at the level of the pancreas and liver, where it may enhance insulin secretion, improve glucose metabolism, and reduce oxidative stress.

### 6.1. Effects on Hyperglycemia

Several studies have shown that consumption of CM by patients with type 1 diabetes can lead to a significant reduction in blood glucose levels, along with a decrease in exogenous insulin requirements by up to 30% [74]. These observations suggest that CM may provide insulin-like proteins or other bioactive compounds that support glycemic control, although the precise mechanisms remain unclear. The oral administration of insulin or protein-based therapeutics is generally challenging, as proteins are susceptible to enzymatic degradation in the gastrointestinal tract, which can disrupt their secondary, tertiary, or quaternary structures and limit their intestinal absorption [75]. Interestingly, CM exhibits physicochemical properties that may help preserve protein integrity during digestion. Specifically, it does not coagulate under low pH conditions and exhibits higher buffering capacity compared to milk from other ruminants [76], potentially reducing protein denaturation in the stomach and enhancing the bioavailability of functional peptides. Moreover, CM contains small-sized immunoglobulins that may contribute to immune modulation and help preserve pancreatic β-cell populations by downregulating autoimmune responses [77,78].

Different mechanisms have been proposed to explain the antidiabetic effects of CM, including: (1) the encapsulation of insulin-like proteins in lipid vesicles, which may enhance their stability and targeted delivery [79]; (2) a higher concentration of insulin-like proteins compared to bovine milk (52 mU/mL vs. 16.32 mU/mL, respectively); and (3) a greater zinc content, which is essential for pancreatic β-cell function and insulin secretion [48].

The hypoglycemic effects of CM and its bioactive components have been demonstrated in experimental animal models of diabetes. Agrawal et al. [76] reported a significant reduction in blood glucose levels in streptozotocin (STZ)-induced diabetic rats following daily administration of raw CM for four weeks, with glucose levels decreasing from 189.68 ± 28.7 mg/dL to 81.54 ± 11.4 mg/dL. Similarly, Al-Numair et al. [80] showed that daily intake of 250 mL of raw CM over 45 days significantly reduced plasma glucose concentrations in STZ-induced diabetic rats, from 292.38 ± 19.20 mg/dL to 141.57 ± 12.82 mg/dL. Mohieldein et al. [81] further demonstrated that a 30-day treatment with CM in STZ-induced diabetic rats not only reduced blood glucose, urea, uric acid, and creatinine levels but also improved liver function markers, serum albumin, the albumin/globulin ratio, and restored lipid profiles toward normal values. Beneficial effects were also observed with CM-derived bioactive fractions. STZ-induced diabetic mice treated with CM whey protein exhibited a significant reduction in blood glucose levels, from 411 ± 37 mg/dL to 261 ± 25.5 mg/dL after two weeks of treatment [82].

Non-rodent models provided similar evidence: Sboui et al. [83] investigated the effect of CM in alloxan-induced diabetic dogs and reported a marked reduction in blood glucose after five weeks of treatment (500 mL/day), from 10.88 ± 0.55 mmol/L (196.04 ± 9.91 mg/dL) to 5.77 ± 0.44 mmol/L (103.96 ± 7.93 mg/dL).

In a recent study, Arain et al. [84] reported the beneficial effects of CM on several metabolic parameters associated with DM in STZ-induced diabetic rabbits. Treatment with CM alone (100 mL/rabbit/day) or in combination with a reduced insulin dose (50 mL CM + insulin at 3 U/kg/day) significantly lowered blood glucose levels. These promising results may be attributed to the enhancement of the antioxidant defense system, as CM-treated rabbits exhibited higher blood concentrations of superoxide dismutase (SOD), catalase (CAT), and glutathione peroxidase (GPx), along with lower levels of malondialdehyde (MDA), compared to untreated diabetic rabbits.

Supporting the potential role of CM as a functional dietary adjunct in diabetes management, numerous clinical human studies have examined its short- and long-term effects in individuals with both T1DM and T2DM. These studies consistently reported improved glycemic control and reduced reliance on exogenous insulin, highlighting CM as a promising complementary dietary intervention in diabetes management.

In patients with T1DM, Agrawal et al. [27] reported that a three-month supplementation with raw CM (500 mL/day), in addition to standard insulin therapy, significantly reduced the required daily insulin dose (from 41.16 ± 10.32 to 30.0 ± 12.06 units/day) along with improved glycemic control as reflected by decreased HbA1c levels. A follow-up study by the same group [77] extended the supplementation to 12 months and observed further reductions in fasting plasma glucose (from 115.6 ± 14.5 to 100.2 ± 17.4 mg/dL) and insulin requirement (from 30.4 ± 11.97 to 19.12 ± 13.39 units/day), supporting the potential of CM to improve metabolic regulation in T1DM.

Long-term benefits were also reported by Mohamad et al. [85], who supplemented T1DM patients with 500 mL/day of CM over 16 weeks. Fasting blood glucose (FBG) decreased dramatically (from 227.2 ± 17.7 to 98.9 ± 16.2 mg/dL), and insulin requirements were halved (from 48.1 ± 6.95 to 23 ± 4.05 units/day).

In T2DM patients, CM has shown similar beneficial effects. A randomized controlled trial in Iran by Fallah et al. [86] examined the co-administration of long-acting insulin with either 500 mL/day of raw CM or cow milk over a three-month period. By the end of the trial, patients receiving raw CM showed a significantly greater reduction in fasting blood glucose compared to baseline (−20.9 ± 36.1 mg/dL). While both groups experienced reductions in HbA1c, the decrease was more pronounced in the CM group compared to the control group (−3.0 ± 2.0% vs. −1.9 ± 2.6%). No significant differences between groups were observed in fasting insulin levels, insulin resistance (HOMA-IR), or fasting glucose. However, the reduction in daily insulin requirement was significantly greater in the CM group (−4.26 ± 1.62 units) than in the control group (−0.11 ± 0.94 units), suggesting a potential insulin-sparing effect of CM.

Recently, Sboui et al. [87] assessed the effects of a three-month CM supplementation in patients with T2DM and reported significant improvements in both glycemic control and lipid metabolism. Fasting blood glucose levels decreased markedly, from 9.89 ± 0.98 to 6.13 ± 0.55 mmol/L, while postprandial glucose levels dropped from 15.89 ± 4.34 to 7.44 ± 1.02 mmol/L. Moreover, HbA1c levels were reduced by approximately 30%, accompanied by significant reductions in total cholesterol and triglyceride concentrations.

Despite these encouraging findings, the effects of CM on glucose homeostasis are multifaceted and, at times, inconsistent. A comprehensive meta-analysis by AlKurd et al. [88], encompassing 14 randomized controlled trials up to 2021, confirmed significant improvements in glycated hemoglobin (HbA1c) levels and daily insulin dose reduction following CM supplementation in both T1DM and T2DM patients. However, reductions in fasting blood glucose did not reach statistical significance, and no significant changes were observed for fasting serum insulin, postprandial blood glucose, insulin resistance (HOMA-IR), or C-peptide concentrations. Interestingly, T2DM patients appeared to benefit more in terms of fasting blood glucose regulation, whereas T1DM patients experienced greater reductions in HbA1c. Additionally, the form in which CM was consumed, whether fresh, fermented, or pasteurized, did not significantly influence its effectiveness in lowering HbA1c levels [88]. The meta-analytic results regarding the effects of CM on fasting blood glucose, HbA1c. and insulin dose in T1DM and T2DM patients are reported in Table 6.

Finally, in relation to the prevalence of DM, Agrawal et al. [74] conducted a population-based study in northwestern Rajasthan, India, to investigate the association between regular consumption of CM and the prevalence of DM. The study involved approximately 2000 individuals from the Raica community, a population with a long-standing tradition of CM consumption. The findings revealed a markedly lower prevalence of DM among regular CM consumers (0.4%) compared to non-consumers from the same region (5.5%). Logistic regression analysis identified CM consumption as the most significant protective factor against diabetes (Odds Ratio: 12.135, *p* < 0.001), followed by community affiliation (Odds Ratio: 6.537, *p* = 0.001) and lifestyle factors (Odds Ratio: 1.788, *p* = 0.036). Moreover, CM consumers exhibited lower rates of impaired fasting glucose (3.2%) and impaired glucose tolerance (0.8%), supporting the potential role of CM in the prevention of glucose metabolism disorders. The essential features and results about blood plasma glucose and glycated hemoglobin reported by the studies cited in the present section are summarized in Table 7 These findings can, at least partially, confirm the usefulness of CM in supporting conventional therapies for both T1DM and T2DM, and may open avenues for the development of novel hypoglycemic agents. However, most studies are limited by small sample sizes, short intervention periods, and, in several cases, a lack of blinding or randomized design, which necessitates cautious interpretation and further well-controlled research.

### 6.2. Effects on Hyperlipidemia

Dyslipidemia is a frequent comorbidity in all forms of diabetes mellitus, characterized by abnormal plasma lipid profiles that increase the risk of cardiovascular and other vascular complications. Emerging evidence from both experimental animal models and clinical studies suggests that camel milk (CM) may positively influence lipid metabolism, thereby supporting cardiometabolic health in diabetic individuals. These benefits are partly attributed to its distinctive fatty acid profile: CM contains higher levels of long-chain polyunsaturated fatty acids, essential fatty acids (linoleic and linolenic acids), and conjugated linoleic acid (CLA) compared to bovine milk, while being lower in cholesterol and saturated fats. Such a composition may help normalize lipid profiles, lower triglycerides, and improve the LDL/HDL ratio, ultimately reducing cardiovascular risk. Furthermore, its medium-chain fatty acids and elevated L-carnitine content may promote fatty acid oxidation and enhance energy metabolism, offering additional support for glycemic control and overall metabolic health [50].

In an experimental study on alloxan-induced diabetic dogs, Sboui et al. [83] demonstrated that CM supplementation led to a significant reduction in plasma total cholesterol levels, decreasing from 6.17 ± 0.15 mmol/L (239 ± 6 mg/dL) to 4.85 ± 0.61 mmol/L (168 ± 24 mg/dL). Conversely, diabetic control dogs supplemented with cow milk exhibited an increase in total cholesterol from 5.99 ± 0.58 mmol/L (232 ± 22 mg/dL) to 7.13 ± 1.25 mmol/L (276 ± 48 mg/dL). Similarly, AlNumair et al. [80] reported that daily administration of CM (250 mL/day for 45 days) to STZ-induced diabetic rats significantly reduced plasma levels of triacylglycerols, phospholipids, total cholesterol, low-density lipoprotein cholesterol (LDL-C), and very low-density lipoprotein cholesterol (VLDL-C), while concurrently increasing high-density lipoprotein cholesterol (HDL-C). These lipid-modulating effects were corroborated by Khan et al. [81], who observed similar improvements in lipid profiles in diabetic rats receiving fresh CM supplementation.

In human studies, Agrawal et al. [89] observed that six months of CM supplementation in T1DM patients significantly decreased plasma triglycerides (from 92.76 ± 0.18 mg/dL to 31.5 ± 0.17 mg/dL) and LDL-C levels (from 65.18 ± 5.08 mg/dL to 45.54 ± 0.10 mg/dL). However, an earlier study by the same group [27] with a shorter duration of three months did not detect statistically significant changes in lipid parameters, suggesting the importance of longer-term intervention. El-Sayed [90] evaluated a combined treatment of insulin and camel milk in diabetic men, reporting a pronounced reduction in total cholesterol and triglycerides (~45%) and LDL-C (~30%), alongside a significant increase in HDL-C, compared to insulin treatment alone, which resulted in more modest lipid reductions. More recently, Sboui et al. [83] reported a significant decline in triglyceride levels in T2DM patients following a three-month CM supplementation, with values decreasing from 2.2 ± 0.3 mmol/L to 1.59 ± 0.37 mmol/L from the first month onwards.

A meta-analysis regarding the lipid profile in diabetic patients has been performed by Khalid et al. [91]: although a limited number of studies has been involved, it seems that the treatment with CM can reduce the levels of blood triglycerides, cholesterol, and low-density lipoprotein, paralleled by an increase in high-density lipoprotein concentration. The summary results of the meta-analytic process are resumed in Table 8.

### 6.3. Effects on Diabetes Complications

Chronic hyperglycemia in DM leads to dysfunction across multiple organ systems due to the central role of glucose in overall metabolism. Beyond glycemic control, CM has demonstrated potential in mitigating several DM-associated complications.

Several studies have highlighted the protective effects of CM and its whey components on liver function. For example, Hamad et al. [92] reported significant reductions in liver enzyme activities, specifically aspartate aminotransferase (AST) and alanine aminotransferase (ALT), in STZ-induced diabetic rats treated with CM, indicating improved hepatic function. These findings were corroborated by Khan et al. [81] in a similar diabetic rat model, reinforcing CM’s hepatoprotective properties.

CM also exhibits notable antithrombotic effects. Korish et al. [7] demonstrated that CM prevented fibrinogen consumption, modulated platelet function, and prolonged prothrombin time in STZ-induced diabetic rats, collectively indicating strong anticoagulant activity. Notably, these antithrombotic effects were more pronounced in diabetic rats treated with CM compared to those receiving bovine milk, suggesting a unique benefit of CM in managing diabetes-related coagulopathies.

CM has also shown promising effects in mitigating renal complications. In patients with T1DM, CM treatment over a six-month period significantly reduced microalbuminuria, suggesting a nephroprotective role [89]. Similar findings were reported by Mohamad et al. [85], reinforcing the potential of CM in preserving kidney function and delaying the onset of diabetic nephropathy.

Among the most prevalent and challenging complications of both T1DM and T2DM is impaired wound healing. Several studies suggest that CM, particularly its whey proteins and hydrolysates, can promote wound repair through modulation of immune responses and enhancement of tissue regeneration. Badr et al. [82], in an experimental model of STZ-induced diabetic mice treated for one month, demonstrated accelerated wound closure and improved tissue integrity following CM whey supplementation. Notably, the treatment restored hydroxyproline levels, a key amino acid involved in collagen synthesis, indicating enhanced extracellular matrix remodeling and skin regeneration.

Many of CM’s benefits may stem from its ability to counteract oxidative stress, a major factor in vascular and neurological diabetic complications [93]. CM is naturally rich in antioxidant vitamins, particularly vitamin C, which is present at levels approximately three times higher than in cow milk, as well as vitamins A, B2, and E. It also contains essential minerals such as sodium, potassium, copper, magnesium, and zinc [21,94]. This unique nutritional profile contributes to the neutralization of free radicals and the enhancement of the body’s antioxidant defense systems. Supporting this, El-Said et al. [95] reported that CM supplementation in diabetic rabbits significantly reduced oxidative stress markers, including malondialdehyde (MDA), and improved levels of catalase and glutathione, key antioxidant enzymes. Additionally, CM contains bioactive glycoproteins, notably lactoferrin, which possesses anti-inflammatory, antimicrobial, and regenerative properties [96]. Lactoferrin contributes to wound healing by stimulating keratinocyte and fibroblast migration and promoting hyaluronan and collagen synthesis [97]. Its strong affinity for iron ions also allows lactoferrin to function as a bacteriostatic agent, particularly against Gram-negative bacteria [98]. Due to these properties, lactoferrin is being investigated for use in advanced wound dressings, particularly for chronic diabetic wounds [99].

## 7. CM Anti-Diabetic Activity: Proposed Mechanisms of Action

### 7.1. Anti-Hyperglycemic Mechanisms

The hypoglycemic effects of CM are among its most promising therapeutic properties, although the precise molecular mechanisms remain only partially understood. Evidence suggests that CM influences glucose homeostasis through multiple biological pathways, including interactions with cell surface receptors, modulation of gene expression, and the activity of bioactive peptides and proteins. Specifically, CM appears to regulate insulin synthesis and secretion while facilitating glucose transport and metabolism in target tissues. The proposed mechanisms underlying the anti-hyperglycemic effects of CM can be broadly grouped into three main pathways [100]:

(1) Direct modulation of insulin receptors and glucose transport across cell membranes;
(2) Stimulation of insulin secretion by pancreatic β-cells through both direct and indirect mechanisms;
(3) Support of pancreatic β-cell survival and function, thereby enhancing overall pancreatic activity.

**Figure 1:**
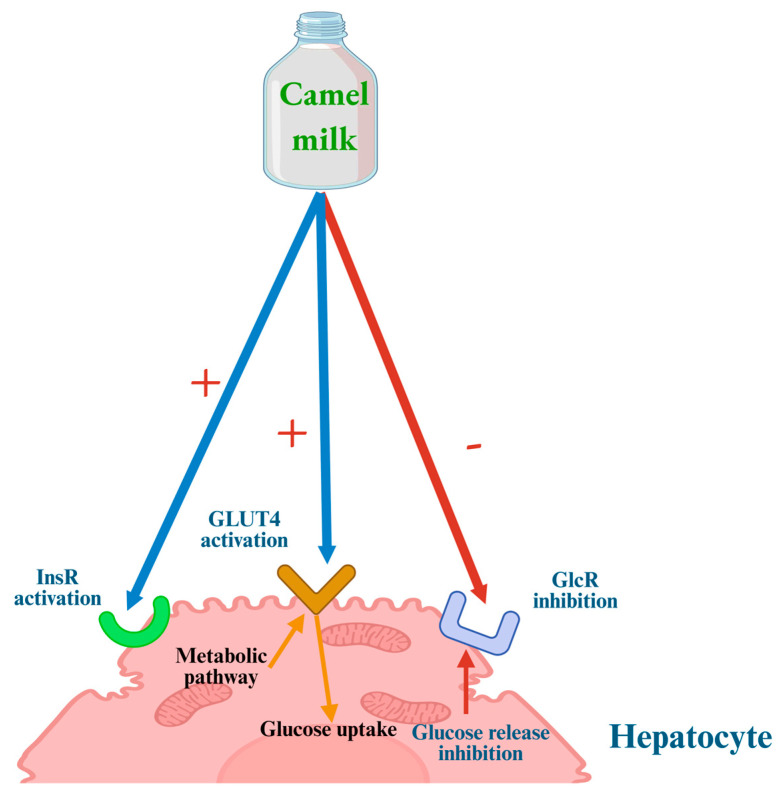
Possible action mechanisms of CM on the liver in glycemic control. InsR: insulin receptor; GLUT4: glucose-specific transporter; GlcR: glucagon receptor (partially redrawn from Ayoub et al. [100]).

Although the anti-diabetic potential of CM is increasingly recognized, confirmation through comprehensive in vivo studies remains necessary. Current evidence indicates that its effects are primarily mediated through coordinated actions on both the pancreas and the liver, supporting glucose regulation and overall metabolic balance. In particular, the liver is a key insulin-sensitive organ that expresses not only insulin receptors but also glucagon receptors (GCGR) [100]. CM may exert hypoglycemic effects through modulation of the hepatic GCGR activity (Figure 1). Evidence suggests that CM can directly enhance the activity of glucose transporter type 4 (GLUT4), thereby increasing hepatic glucose uptake capacity [100]. Moreover, CM whey proteins appear to confer hepatoprotective effects, potentially mitigating liver injury [101]. This protective role is thought to involve the upregulation of key genes involved in insulin signaling and glucose metabolism, including insulin receptor substrate-2 (IRS-2), protein kinase B (AKT), phosphoinositide 3-kinase (PI3K), and glycogen synthase [101].

The hypothesis of a direct interaction between CM and insulin receptors is supported by the high concentration of insulin present in CM (approximately 52 U/L), which is about four times greater than that found in cow’s milk [102]. In addition to insulin, CM contains insulin-like proteins capable of binding to and activating insulin receptors. These bioactive molecules are believed to be protected by specific structural or carrier mechanisms that preserve their integrity in the gastric environment and enhance their intestinal absorption [48,79,103,104]. Notably, CM contains approximately three times more insulin-like proteins than bovine milk [48], a characteristic that further supports the hypothesis of a direct insulin-mimetic effect on target tissues.

Along with its direct effects, CM may exert indirect actions on insulin synthesis and secretion by pancreatic β-cells, potentially mediated by bioactive peptides. These peptides are thought to influence various biochemical pathways involved in the regulation of insulin production and exocytosis [100]. Furthermore, CM may modulate pancreatic function through its interaction with key hormones and regulatory peptides. These include protein hormones such as glucagon, as well as insulinotropic agents like gastric inhibitory polypeptide (GIP) and glucagon-like peptide-1 (GLP-1). Enzymes involved in incretin regulation, particularly dipeptidyl peptidase-IV (DPP-IV), a known inhibitor of GIP and GLP-1, also appear to be influenced by components of CM, thereby enhancing insulin secretion [105,106].

**Figure 2:**
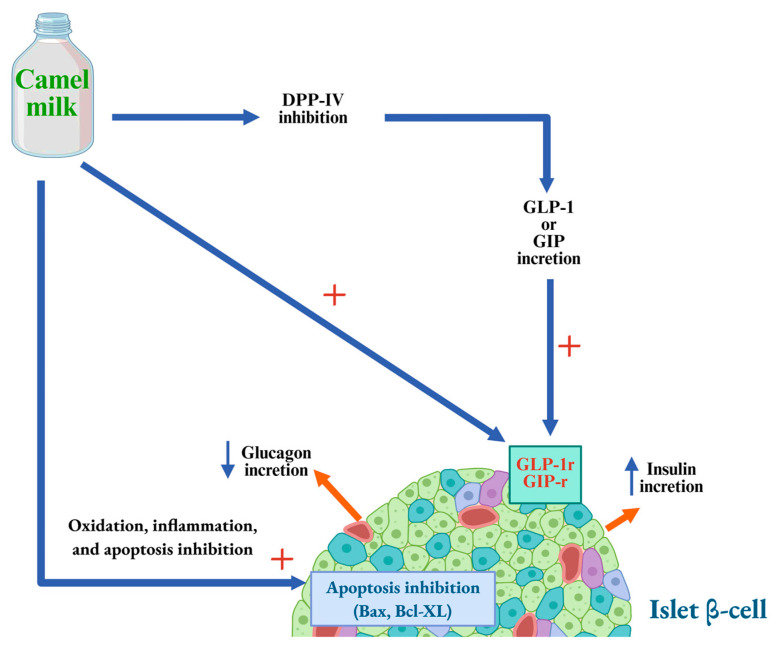
Proposed mechanisms of action of CM on pancreatic β-cells involved in the regulation of insulin and glucagon secretion. DPP-IV: dipeptidyl peptidase-IV; GLP-1: glucagon-like peptide-1; GIP: gastric inhibitor peptide; GLP-1r: GLP-1 receptor; GIPr: GIP receptor; Bax: Bcl-2-like protein 4; Bcl-XL: B-cell lymphoma-extra-large protein (partially redrawn from Ayoub [100]).

The indirect effects of CM on pancreatic function may involve multiple mechanisms, including the stimulation of GLUT4 transporter activity, the suppression of glucagon secretion by pancreatic α-cells, and the inhibition of enzymes such as dipeptidyl peptidase-IV (DPP-IV), a key regulator of incretin degradation (Figure 2). Incretins, such as gastric inhibitory polypeptide (GIP) and glucagon-like peptide-1 (GLP-1), enhance insulin secretion while concurrently suppressing glucagon release from α-cells. Therefore, inhibition of DPP-IV prolongs incretin activity and promotes glycemic control through enhanced insulin release and reduced glucagon levels [100]. Additionally, hydrolyzed CM-derived molecules may exert allosteric modulatory effects on GIP and GLP-1 receptors in pancreatic α-cells, further contributing to their insulinotropic and glucagon-suppressing effects [100]. Abdulrahman et al. [107] demonstrated that CM exerts an allosteric modulation on insulin receptors, facilitating a conformational transition of the activated receptor from ‘active conformation A’ to a more stable and functionally potent active conformation B.’ This shift enhances the efficiency of downstream intracellular signaling pathways.

Finally, CM molecular complexes may exert beneficial effects on the immune system and redox balance across various cell types, including pancreatic β-cells. These properties can enhance β-cell function by improving their overall viability, thereby promoting insulin synthesis and secretion. In type 1 diabetic rat models, administration of CM whey proteins was associated with a significant reduction in pro-inflammatory cytokines such as interleukin-1β (IL-1β), interleukin-6 (IL-6), and tumor necrosis factor-alpha (TNF-α), accompanied by an increase in anti-inflammatory cytokines including interleukin-2 (IL-2) and interleukin-4 (IL-4) [108,109,110]. Moreover, a decrease in lymphocyte apoptosis was observed [108], which appears to be mediated by the downregulation of the pro-apoptotic protein Bax (Bcl-2-associated X protein) and the modulation of the anti-apoptotic protein Bcl-xL (B-cell lymphoma-extra-large protein) expression, as demonstrated in type 1 diabetic rats [110] (Figure 2).

### 7.2. Anti-Lipidemic Mechanisms

The anti-lipidemic effects of CM are closely linked to its glucose-lowering properties. Moneim et al. [111] reported that in streptozotocin (STZ)-induced diabetic rats fed with CM, improvements in lipid profiles were observed, including reductions in serum triglycerides, total cholesterol, low-density lipoprotein (LDL), and very-low-density lipoprotein (VLDL), alongside an increase in high-density lipoprotein (HDL). These lipid improvements were accompanied by an upregulation of the GLUT4 receptor gene expression and an elevation in the gene expression of peroxisome proliferator-activated receptor gamma (PPAR-γ). PPAR-γ plays a crucial role in regulating genes involved in glucose homeostasis and exerts direct effects on pancreatic β-cells and hepatic tissues to maintain glucose balance [112]. Zheng et al. [112] reported that mice fed a high-fat diet and treated with CM endogenous peptides (CMEP) showed reduced gut microbiota disturbances, by decreasing the intestinal permeability through the enhancement of the activity of occludin, the zonula occludens-1 (ZO-1), and claudin proteins in high-fed mice, evidencing the participation of CMEP as active ingredients in supporting diabetes treatment. CMEP can activate the IRS/PI3K/AKT signaling pathway, ameliorating carbohydrate homeostasis.

### 7.3. Antioxidant Mechanisms of CM

T1DM is an autoimmune disorder characterized by the selective destruction of insulin-producing pancreatic β-cells without apparent pathological alterations of other Langerhans cells. T1DM varies in age, severity of autoimmune response, and therapy efficacy, with both humoral and cellular immunity involved in its pathogenesis. The predominant theory is that β-cell pancreatic islets in patients with T1DM suffer from inflammation, leading to a condition called insulitis. Regulatory T cells are also defective in this autoimmune disease setting. Animal models show that CD4+ and CD8+ T cells participate in the development of T1DM, targeting several β-cell auto-antigens and related peptide epitopes. T-cell subtypes can induce destructive peri-islet inflammatory infiltrate and overt diabetes. Macrophages seem to be critical mediators of islet inflammation, due to their ability to secrete cytokines and produce reactive oxygen species (ROS); the biology of the beta cell can therefore directly influence the response to an inflammatory environment, leading to multiple pathways contributing to pancreatic beta cell death. In T2DM, hyperglycemia leads to an increase in ROS production. This heightened oxidative stress can impair the function of pancreatic β-cells; this situation contributes to insulin resistance, making it harder for cells to use insulin to absorb glucose from the blood. Moreover, oxidative stress damages various cellular components, including lipids, proteins, and DNA. This damage is a major contributor to the long-term complications of diabetes. For a complete review of these concepts refer to the dedicated review of Tsalamandris et al. [113].

The antioxidant properties of CM and whey-derived peptide fractions contribute significantly to enhanced wound healing in diabetic rats and mice [114,115]. These effects are linked to enhanced immune cell proliferation and modulation of cytokine profiles, with reductions in pro-inflammatory markers such as tumor necrosis factor-alpha (TNF-α), interleukin-1 (IL-1), and interleukin-6 (IL-6), and an increase in the anti-inflammatory cytokine interleukin-10 (IL-10) [82,115,116,117]. Moreover, CM’s notably high vitamin C content further contributes to ROS neutralization and protects against microvascular complications such as retinopathy and nephropathy [117].

Additionally, insulin naturally present in CM undergoes first-pass metabolism in the liver, where it inhibits hepatic gluconeogenesis and consequently enhances the hypoglycemic action of pancreatic β-cells [118].

Some of these antioxidant and anti-inflammatory properties are also observed in fermented CM products, including shubat (China and Kazakhstan), khoormang (Mongolia), iben (Saudi Arabia), garris (Sudan), suusac (Kenya), and ititu (Ethiopia) [5].

### 7.4. CM Wound Healing Mechanisms

The wound healing properties of CM are attributed to its diverse bioactive components, including immunoglobulins, α-lactalbumin, peptidoglycans, and lactoferrin, which collectively provide antioxidant and regenerative support [119]. This effect was demonstrated in streptozotocin (STZ)-induced diabetic rats treated with CM by gavage, where treated animals exhibited a significant reduction in wound size and accelerated tissue recovery. These outcomes are primarily associated with increased synthesis of hydroxyproline in the affected tissues, indicating enhanced collagen formation, as reported by Badr et al. [82,114]. Moreover, CM treatment in diabetic rats results in elevated levels of key antioxidant enzymes, such as glutathione, superoxide dismutase, and catalase, which protect cells from oxidative damage and facilitate tissue repair [116]. Furthermore, CM positively modulates the expression of several chemokines involved in tissue repair, including macrophage inflammatory protein-1 alpha (MIP-1α), macrophage inflammatory protein-2 (MIP-2), calcitonin gene-related peptide alpha (KC or katacalcin), fractalkine (CX3CL1), transforming growth factor-beta (TGF-β), and periplakin (PPL) [100]. Additionally, the inhibitory activity of CM on dipeptidyl peptidase-IV (DPP-IV) is evident in wounded tissues, further supporting and accelerating the healing process.

### 7.5. Hepatoprotective Mechanisms of CM

CM has been shown to protect against various liver injuries, including both alcoholic and non-alcoholic hepatitis, largely due to the activity of its immunoglobulins [120]. For instance, Dou et al. [101] reported that a specific CM-derived whey protein, camel whey protein 8 (CWP8), exerts protective effects in insulin-resistant HepG2 cells by modulating the PI3K/AKT (phosphoinositide 3-kinase/protein kinase B) signaling pathway and reducing oxidative stress.

### 7.6. CM Kidney Protective Mechanisms

In diabetes, metabolic dysregulation often elevates endotoxin levels in the kidney, which activate toll-like receptors (TLRs) and trigger pro-inflammatory cascades. Among these, TLR-4 plays a key role by stimulating nuclear factor kappa B (NF-κB) and promoting the release of potent cytokines such as interleukin-1β (IL-1β), interleukin-6 (IL-6), and tumor necrosis factor-α (TNF-α) [121]. CM proteins and their bioactive derivatives have been shown to modulate the TLR-4/MAPK (mitogen-activated protein kinase)/NF-κB signaling pathway, thereby attenuating inflammation and enhancing renal protection [122]. Additionally, CM supports kidney function through activation of the phosphoinositide 3-kinase/protein kinase B/endothelial nitric oxide synthase (PI3K/AKT/eNOS) pathway, which contributes to vascular health within the renal system [123]. Both whole CM and its exosomes have demonstrated efficacy in reducing diabetic nephropathy in rat models by downregulating key nephropathy-associated genes, including transforming growth factor-β1 (TGF-β1), integrin subunit beta-2, kidney injury molecule-1 (KIM-1), and intercellular adhesion molecule (ICAM) [124].

### 7.7. The Role of Lactoferrin from CM

Lactoferrin is one of the most bioactive proteins in camel milk and a key contributor to its anti-diabetic effects. In vitro studies using immortalized cell lines such as HEK293 and HepG2 have shown that lactoferrin modulates insulin receptor activity and regulates the extracellular signal-regulated kinase (ERK) and protein kinase B (AKT) signaling pathways [125]. Its hypoglycemic action operates through two main mechanisms. First, it activates the insulin receptor/AKT (IR/AKT) hypoglycemic pathway, enhancing the expression and function of glucose transporter (GLUT) proteins. Additionally, lactoferrin enhances both the efficacy (increased Emax) and potency (decreased EC50) of insulin binding to its receptor [126]. Second, lactoferrin inhibits the TLR/AKT/NF-κB signaling pathway, exerting an anti-inflammatory effect that may contribute to the restoration of pancreatic β-cell structure and function [127].

Evidence also suggests that lactoferrin, along with intact whey proteins and their hydrolysates, enhances insulin receptor activity in vitro, primarily through positive allosteric interactions [126,127]. Moreover, Ayoub et al. [128] suggest that intact CM proteins or their fractions may also bind to other membrane receptors, which in turn could allosterically potentiate insulin receptor activity.

### 7.8. The Role of Protein-Derived Peptides

The beneficial effects of milk proteins on hyperglycemia in both T1DM and T2DM are significantly enhanced by bioactive peptides generated during gastric and intestinal digestion [129]. These peptides contribute directly or indirectly to blood glucose regulation and metabolic control, as demonstrated by several studies [130,131]. The underlying mechanisms likely originate in the intestine, where CM proteins inhibit key digestive enzymes such as intestinal α-glucosidase and pancreatic α-amylase, which are responsible for carbohydrate breakdown and glucose release [132]. Additionally, peptides derived from CM can inhibit dipeptidyl peptidase-IV (DPP-IV), an enzyme that degrades incretins, thereby enhancing insulin activity and improving glycemic control [133,134].

Antidiabetic peptides from CM have been obtained using various hydrolytic methods, including bacterial fermentation and simulated gastrointestinal digestion [134]. Lactic acid bacteria, particularly *Streptococcus thermophilus* and *Lactobacillus bulgaricus*, have been extensively used to hydrolyze milk proteins, often in combination with probiotic strains such as *Lactobacillus acidophilus*, *Lactobacillus casei*, and *Bifidobacterium bifidum*. These microorganisms contribute a range of enzymatic activities, including proteases and glycosidases, which facilitate the release of bioactive peptides.

Interestingly, the bioactivity of processed CM increases with storage time; its enzyme-inhibitory capacity becomes more pronounced between 3 and 6 h post-processing. In contrast, cow milk does not exhibit similar improvement under identical conditions.

The first two peptides isolated from CM with confirmed human pancreatic α-amylase inhibitory activity were obtained through fermentation with *Lactiplantibacillus plantarum*, and their sequences were: Thr-Asp-Val-Met-Pro-Gln-Trp-Trp and Met-Lys-Phe-Phe-Ile-Phe-Thr-Cys-Leu-Leu-Ala-Val-Val-Leu-Ala-Lys [135]. Notably, these peptides demonstrated higher inhibitory activity compared to those derived from cow milk [136].

Numerous studies have reported the identification of bioactive, protein-inhibitory peptides generated through in vitro proteolysis of CM proteins, simulating gastrointestinal digestion [134]. These enzymatic processes are inherently complex, as their efficiency and outcomes are highly dependent on factors such as temperature, pH, enzyme concentration, and hydrolysis duration. To date, various proteolytic enzymes, including alcalase, papain, and bromelain, have been employed to release peptides with antidiabetic properties, although their effectiveness varies according to the enzyme used and the reaction conditions [101,129]. Hydrolysis of whole camel milk proteins by alcalase produces more potent pancreatic α-amylase inhibitory peptides compared to papain or bromelain [137]. Notably, alcalase-derived peptides such as Lys-Phe-Gln-Trp-Gly-Tyr and Lys-Asp-Leu-Trp-Asp-Phe-Lys-Gly-Leu, along with the bromelain-derived peptide Met-Pro-Ser-Lys-Pro-Pro-Leu-Leu, bind to nine key residues within the active site of pancreatic α-amylase. Additionally, the peptide Gly-Met-Ala-Gly-Gly-Pro-Pro-Leu-Leu interacts with eight active site residues, while other peptides—Ala-Glu-Trp-Leu-His-Asp-Trp-Lys-Leu, Ser-Gln-Asp-Trp-Ser-Phe-Tyr, and Trp-Asn-Trp-Gly-Trp-Leu-Leu-Trp-Gln-Leu—bind to seven critical residues of the enzyme [137].

The inhibitory activity on DPP-IV appears enhanced following alcalase hydrolysis of CM proteins. Two peptides, Asp-Asn-Leu-Met-Pro-Gln-Phe-Met and Trp-Asn-Trp-Gly-Trp-Leu-Leu-Trp-Gln, bind to eight and seven active sites of DPP-IV, respectively, while three others—Cys-Phe-Leu-Pro-Leu-Pro-Leu-Leu-Lys, Met-Met-His-Asp-Phe-Leu-Thr-Cys-Leu-Met, and Ser-Gln-Asp-Trp-Phe-Ser-Tyr—interact with six active residues [137]. Additional inhibitory oligopeptides, such as Leu-Pro-Val-Pro-Gln, Val-Leu, and Leu-Pro-Gln, have also been reported to act on DPP-IV following CM proteolysis [138].

Mudgil et al. [136] further investigated the hydrolysis of CM casein proteins by alcalase and pronase E, observing that both enzymes exhibit similar inhibitory effects on pancreatic α-amylase and intestinal α-glucosidase. The most potent α-amylase inhibitory peptides identified were Phe-Leu-Trp-Pro-Glu-Tyr-Gly-Ala-Leu, Asp-Gly-Ala-Leu-His-Pro-Pro-Leu, and Ala-Gly-Cys-Pro. Moreover, the peptide His-Ala-Ser-Trp-Pro-Leu-Leu demonstrated inhibitory activity against DPP-IV by binding to five residues, suggesting a potential allosteric inhibition mechanism [132].

Some of the beneficial effects of CM on glycemic control may be mediated by insulin-like small molecules found broadly in animals and plants. Additionally, certain milk fatty acids, including trans fatty acids, can influence glycemic regulation either positively or negatively. For example, trans-palmitoleate (trans-16:1n-7 palmitoleate) has been associated with a reduced incidence of diabetes, whereas its *cis* isomer, *cis*-palmitoleate, also improves glycemia but may increase cardiovascular risk in humans [138].

CM proteins, and particularly their derived peptides, also exhibit angiotensin-converting enzyme (ACE) inhibitory activity. ACE is an extrinsic membrane metalloprotease that plays a crucial role in the renin-angiotensin system by converting angiotensin I into angiotensin II, a potent vasoconstrictor that promotes hypertension [139]. Several ACE-inhibitory peptides have been isolated from bovine milk trypsin hydrolysates. Maruyama et al. [140] identified peptides such as CEI12 (Phe-Phe-Val-Ala-Pro-Glu-Pro-Glu-Val-Phe-Gly-Lys), CEI7 (Ala-Val-Pro-Tyr-Pro-Gln-Arg), and CEI5 (Phe-Phe-Val-Ala-Pro), which demonstrated ACE inhibition in rat models.

Anwar et al. [141] confirmed the crucial role of CM-derived peptides in glycemic control through in silico and in vitro studies with synthetic CM peptides, underlining that the studied peptides could represent the “primary backbone to develop more potent and rationale CM-derived peptides” in glycemic control protocols.

### 7.9. Camel Milk Exosomes: A Novel Nanocomponent in Glycemic Control and Diabetes Management

Exosomes are small extracellular nanovesicles, typically 40–100 nm in diameter, released by various cell types, including those that produce milk. These vesicles carry diverse bioactive molecules, such as proteins, lipids, and nucleic acids, and have been detected in multiple body fluids, including blood, urine, and milk. Milk-derived exosomes play a critical role in intercellular communication by transferring key molecules like DNA, mRNA, microRNAs, proteins, and peptides between cells, thereby influencing physiological processes [142].

Exosomes in milk have also been shown to have potential therapeutic applications, since their multiple properties (anti-microbial, anti-viral, anti-parasitic, anti-inflammatory, antioxidant, anti-allergic, and immunomodulatory) [143,144,145]. They could serve as a delivery system for bioactive molecules, such as drugs, to target cells or tissues. This has led to research exploring the use of exosomes derived from milk as a vehicle for drug delivery in various diseases.

The beneficial effects of exosomes are often attributed to their rich phospholipid composition. In CM, for example, phosphatidylserine and phosphatidylcholine concentrations range from approximately 10–15 mg/mL and 20–25 mg/mL, respectively, remaining relatively stable throughout lactation [145,146]. These lipid components likely contribute to the therapeutic potential of camel milk exosomes in glycemic regulation and the mitigation of diabetes-related complications.

Exosomes originate from multivesicular bodies within cells and are released through membrane fusion, distinguishing them from larger microvesicles, which measure approximately 1 µm in diameter [147]. Their small size and natural capacity to carry diverse molecular cargo make exosomes promising candidates for targeted delivery systems, including pharmacological applications. The diverse molecular cargo within milk exosomes supports their multifaceted biological functions. They carry proteins like lactoferrin and κ-casein, as well as adhesion molecules such as CD9, CD63, and CD81, which belong to the tetraspanin family [145]. Camel colostrum exosomes exhibit strong immune-stimulatory effects, enhancing neonatal immune responses [148]. Additionally, the robust phospholipid bilayer encapsulating exosomes protects them from degradation by gastric acid and digestive enzymes, ensuring their stability during gastrointestinal transit [149].

CM exosomes display notable anti-inflammatory activity by reducing pro-inflammatory cytokines such as TNF-α, TGF-β1, IL-1β, and NF-κB in tumor cells [150,151]. Ibrahim et al. [152] further reported that camel milk exosomes suppress TNF-α and IL-6 expression and decrease gamma-interferon (IFN-γ) levels, thereby mitigating tumor progression. Supporting this, CM administration also downregulates TNF-α, IL-1β, and NF-κB expression in a rat model of 5-fluorouracil-induced renal inflammation [153], while CM whey proteins reduce inflammatory markers in diabetic rats [154].

The immunomodulatory effects of CM exosomes involve complex interactions among natural killer (NK) cells, CD4+ helper T cells, and CD8+ cytotoxic T cells [155]. This mechanism was demonstrated in vivo by Badawy et al. [156] in rat spleen and supported by Laghi et al. [157] in cancer patients, who observed improved survival rates linked to enhanced CD8+ T cell activity. These cytotoxic T cells can also indirectly inhibit tumor growth by stimulating additional cytokine expression [154]. Notably, whole camel milk induces stronger increases in NK, CD4+, and CD8+ cells than isolated milk exosomes, likely due to supplemental immunomodulatory components such as immunoglobulins, lysozyme, lactoferrin, lactoperoxidase, and caseins [158].

Furthermore, camel milk contains heavy chain-only antibodies (nanobodies) capable of crossing cell membranes and triggering immune responses [144]. Camel milk-derived exosomes also exert antioxidant effects by enhancing catalase, glutathione peroxidase, and superoxide dismutase activities, while reducing lipid peroxidation (measured as malondialdehyde) and inducible nitric oxide synthase (iNOS) expression [156]. Ayoub et al. [128] underline that CM casein hydrolysate has antidiabetic effects. They found that CM contains peculiar proteins and peptides that help manage blood sugar. CM works in two main ways to combat diabetes: first, CM appears to protect and improve the function of pancreatic β-cells, helping the incretion of the appropriate amount of insulin. Second, CM improves cell response to insulin, which improves glucose use and processing glucose. These CM components may work by uniquely influencing the insulin receptor, helping cells take in more glucose from the bloodstream. All this research provides strong evidence that CM could benefit diabetes management.

### 7.10. Non-Parenteral Administration of Insulin: Looking Forward

The non-parenteral administration of insulin in human patients remains a significant challenge due to the risk of severe hypoglycemia. Among alternative routes, oral administration is particularly appealing; however, insulin, being a protein, is readily degraded in the acidic and enzymatic environment of the stomach, limiting its bioavailability and therapeutic efficacy.

To enhance insulin stability in the gastrointestinal tract and improve its bioavailability, several oral nanodelivery systems have been developed. These nanoformulations can be customized to individual patient needs, offer prolonged action, and allow controlled release of the therapeutic molecule [159]. Unlike the milk of other mammals, CM protects insulin from degradation by gastric hydrochloric acid and proteolytic enzymes. Notably, the lipid micelles in CM are larger than those found in other mammalian milks [160], a characteristic that may confer a protective effect by encapsulating insulin and other labile proteins, facilitating their transit through the stomach [79]. This hypothesis is supported experimentally by Prego et al. [161], who studied protein release from chitosan-based nanoparticles at the intestinal mucosal level, and by Vila et al. [162], who investigated protein delivery using various polymer nanoparticles.

Additionally, insulin from CM has a smaller molecular size compared to other insulins, which may facilitate its more efficient integration into systemic circulation [163]. Furthermore, camel insulin contains higher levels of zinc compared to other insulins, a characteristic that may enhance its interaction with insulin membrane receptors and potentiate the insulin secretory activity of pancreatic β-cells [104].

### 7.11. Additional Considerations: Potential Limitations, Safety Risks, and Regulatory Status

In recognition of the nutritional, economic, and cultural significance of camelids, the United Nations designated 2024 as the “International Year of Camelids”. CM, traditionally consumed in arid and semi-arid regions, is increasingly marketed globally as a premium commodity due to its distinctive nutritional composition, particularly the absence of β-lactoglobulin, a major bovine milk allergen, rendering it closer to human milk and potentially suitable for some individuals with cow’s milk allergy [164]. Although generally associated with lower allergenic potential than bovine milk, CM can still trigger hypersensitivity reactions in susceptible individuals, as it contains proteins such as casein that may act as allergens, and cross-reactivity has been reported in a minority of cow’s milk–allergic patients [165].

Its unique protein, lipid, and vitamin profile underscores its high market value but also presents technological challenges. Low κ-casein content, large casein micelles, and reduced thermal stability lead to poor coagulation, prolonged fermentation, and instability during ultra-high temperature (UHT) processing; pasteurization above 80 °C can cause separation and quality deterioration [6,166].

From a food safety perspective, unpasteurized CM shares the microbiological hazards of other raw animal milks, including *Brucella* spp., *Escherichia coli* O157:H7, *Salmonella* spp., *Listeria monocytogenes*, and *Staphylococcus aureus* [167]. Brucellosis, in particular, is a significant zoonotic concern in endemic regions where raw milk consumption is common. Proper pasteurization and cold-chain management are essential to mitigate these risks.

Economic adulteration, especially dilution with bovine milk, further threatens consumer safety, market integrity, and fair trade [6]. These safety and authenticity challenges are compounded by a fragmented regulatory environment: while some producing countries, including Kenya, Morocco, and the United Arab Emirates, have national or regional CM standards, many major producers lack specific regulation. Existing frameworks rarely address authenticity testing or CM’s technological specificities. No internationally harmonized Codex standard currently exists; however, a proposed Codex framework aims to establish authenticity criteria, integrate allergenic and safety considerations, adapt quality guidelines to CM’s unique processing characteristics, and harmonize regulations to protect consumers and support global trade.

## 8. Conclusions

CM may have the potential as a supporting adjunct in the management of hyperglycemia in diabetic patients, thanks to its unique composition and bioactive properties. Although its production and availability remain limited, further experimental research is crucial to elucidate the mechanisms underlying the hypoglycemic effects of CM and to support its development as a natural therapeutic agent. Additionally, the bioactive compounds derived from CM, given their antioxidant potential and positive effects on key metabolic organs such as the liver, pancreas, and kidneys, may have broader therapeutic applications in various chronic and metabolic diseases. Moreover, regular consumption of CM may also support metabolic homeostasis in healthy individuals, contributing to the promotion of overall health and disease prevention. It is desirable to extend clinical trials to larger groups of patients and with more complex experimental designs, with the aim of reinforcing long-established hypotheses about the positive aspects of using CM in different types of diabetes mellitus. CM may thus have great scope for expansion, due to its peculiar composition and a growing world-wide demand, as pointed out by Sharma et al. [9]: by taking advantage of the SWOT framework (Strengths, Weaknesses, Opportunities, Threats) for industries, CM can greatly amplify its production and market possibilities, fostering the use of a potentially valuable resource for millions of people worldwide.

## Figures and Tables

**Table 1:** Raw Camel Milk production in 2022 (in tons) [14].

Country	Production (tons)
Kenya	1,096,698
Somalia	987,842.9
Pakistan	944,000
Mali	294,248.6
Ethiopia	220,446
Saudi Arabia	135,540
Niger	106,597.4
United Arab Emirates	79,434.44

**Table 3:** Descriptive, resumptive statistics (*n* = 82) for key compositional parameters of camel milk (Bactrian and dromedary), compiled from literature data (Konuspayeva et al. [33]), and recalculated by the authors.

						Percentile	
Variable	Mean	Median	Std Dev	Min	Max	2.5th	97.5th
Fat (g/dL)	3.82	3.70	1.08	0.28	6.40	2.30	5.60
Total protein (g/dL)	3.36	3.30	0.63	2.15	4.90	2.28	4.61
Lipids (g/dL)	4.55	4.60	0.69	2.40	5.80	3.26	5.80
Dry matter (g/dL)	12.51	12.73	1.61	8.64	16.08	9.03	15.52
Ash (g/dL)	0.78	0.80	0.09	0.60	1.05	0.60	0.98

**Table 4:** Mean value [95% confidence interval] values of dromedary and Bactrian camel milk components. The data reported are calculated after a meta-analytical procedure [65]. *p*-value reports the significance of between-specie differences; n.s.—*p*-value ≤ 0.05. n.r.—not reported.

Parameter	*Camelus dromedarius*	*Camelus bactrianus*	*p*-Value
Total Protein (%)	3.10 [3.01; 3.20]	3.92 [3.67; 4.17]	0.0001
Fat (%)	3.34 [3.23; 3.44]	5.49 [5.02; 5.96]	0.0001
Lactose (%)	4.33 [4.21; 4.45]	4.80 [4.13; 5.47]	n.s.
Ash (%)	0.77 [0.75; 0.79]	0.86 [0.82; 0.90]	0.0003
Total solids	11.34 [10.93; 11.75]	11.00 [9.62; 12.37]	n.s.
Ca (%) mg/100 g	111.31 [104.97; 117.65]	141.60 [117.87; 165.32]	0.02
Fe (%) mg/100 g	0.46 [0.19; 0.72]	0.21 [0.17; 0.25]	n.s.
K mg/100 g	113.34 [94.31; 132.36]	191.0 [189.70; 192.30]	0.0001
Mg mg/100 g	9.65 [7.92; 11.39]	n.r.	n.r.
Na mg/100 g	48.74 [39.66; 59.89]	n.r.	n.r.
Zn mg/100 g	1.68 [1.45; 1.91]	n.r.	n.r.
Vit C (Ascorbic acid) mg/100 g	5.22 [4.61; 5.83]	10.26 [−4.18; 24.70]	n.s.
Vit A (Retinol) mg/100 g	0.43 [0.05; 0.81]	0.10 [0.09; 0.1]	n.s.
Vit B1 (Thiamine) mg/100 g	0.06 [0.05; 0.08]	0.01 [0.01; 0.013]	0.0001
Vit B2 (Riboflavin) mg/100 g	0.13 [0.05; 0.21]	0.12 [0.10; 0.15]	n.s.
Vit B3 (Niacin) mg/100 g	0.51 [0.42; 0.59]	n.r.	n.r.
Vit B6 (Pyridoxine) mg/100 g	0.14 [0.09; 0.18]	0.05 [0.05; 0.06]	0.0006
Vit B12 (Cyanocobalamin) mg/100 g	0.0039 [0.0015; 0.0064]	n.r.	n.r.

**Table 5:** Mean composition of dromedary colostrum. Table readapted from Konuspayeva et al. [66]. Some variables are excluded from the original work (SD: Standard deviation).

Parameters	Mean ± SD	Min	Max
Total protein, %	6.03 ± 4.70	3.19	17.20
Fat, %	7.88 ± 8.23	1.56	25.94
Lactose, %	3.63	–	–
Ca ^1^, g/L	0.589 ± 0.700	0.104	1.877
P ^2^, g/L	0.404 ± 0.438	0.083	1.030
Fe ^3^, mg/L	2.50 ± 0.97	1.20	3.70

^1^ Ca: calcium; ^2^ P: phosphorus; ^3^ Fe: iron.

**Table 6:** Meta-analytic results for fasting blood glucose (FBG) and glycated hemoglobin (HbA1c) expressed as mean difference and 95% confidence interval (95%CI) for human patients treated with camel milk vs. control patients [88].

Group/Subgroup	Studies Involved	Total Patients	Parameter	Estimated Mean Difference [95% CI]	*p*-Value
DM (total)	15	641	FBG (mg/dL)	−23.32 [−47.33, 0.70]	0.06
DM (total)	12	585	HbA1c (%)	−1.24 [−2.00, −0.48]	0.001
DM (total)	7	214	Insulin dose (%)	−16.72 [−22.09, −11.35]	<0.0001
Type 1 DM	7	217	FBG (mg/dL)	−27.20 [−73.97, 19.57]	0.25
Type 1 DM	7	217	HbA1c (%)	−1.21 [−2.24, −0.19]	0.02
Type 2 DM	8	400	FBG (mg/dL)	−15.62 [−26.71, −4.54]	0.006
Type 2 DM	5	368	HbA1c (%)	−1.27 [−2.53, 0.00]	0.05

**Table 7:** Study characteristics regarding the effects of CM consumption on fasting blood glucose (FBG) and glycated hemoglobin (HbA1c). The references are cited in the “Effects on hyperglycemia” paragraph.

Author(s), Year	Specie	Diabetogen	Total n. of Subjects	Milk Dosage	Treatment time	Effects on FBG or Hb1Ac (if Indicated)	*p*-Value
Agrawal et al., 2003	Human	T1DM	24	500 mL/d (randomized)	3 mo.	118.16 ± 7.15 mg/dL (control) 100 ± 16.2 ±mg/dL (treated)	<0.001
Agrawal et al., 2004	Rat	STZ	32	250 mL/d/head CM Vs. 250 mL/d/head cow milk	3 wk	191.33 ± 7.46 mg/dL (Cow milk) 86.25 ± 12.77 mg/dL (CM)	<0.05
Agrawal et al., 2007	Human	T1DM	50	500 mL/d	12 mo.	104.00 ± 15.87 mg/dL (control) 100.20 ± 17.40 mg/dL (treated)	0.002
Mohamad et al., 2009	Human	T1DM	54	500 mL/d (randomized controlled)	16 wk	FBG 227.2 ± 17.7 mg/dL (control) 98.9 ± 16.2 mg/dL (treated) HbA1c 9.59 ± 2.05 % (control) 7.16 ± 1.84 % (treated)	<0.05 <0.05
Al Numair et al., 2011	Rat	STZ	30	250 mL/d/head	45 d	292.38 ± 19.20 mg/dL (before) 141.57 ± 12.82 (after)	<0.05
Badr, 2013	Mouse	STZ	30	100 mg whey protein/kg b.w.	13 d	373.6 ± 32 mg/dL (control) 261 ± 25.5 mg/dL (treated)	<0.005
Mohieldein et al., 2013	Rat	STZ	20	400 mL/d/cage	30 d	520.46 ± 8.90 mg/dL (control) 235.61 ± 7.10 mg/dL (treated)	<0.05
Badr, 2013	Mouse	STZ	30	100 mg whey protein/kg b.w.	13 d	373.6 ± 32 mg/dL (control) 261 ± 25.5 mg/dL (treated)	<0.005
Fallah et al., 2020	Human	T2DM	40	500 mL/d (randomized controlled)	3 mo.	FBG 169.3 ± 78.9 mg/dL (before treat.) 148.4 ± 59.5 mg/dL (after treat.) HbA1c 12.7 ± 2.6% (before treat.) 9.4 ± 0.3% (after treat.)	0.02 0.001
Sboui et al., 2022	Human	T2DM	60	500 mL/d	3 mo.	8.37 ± 0.79 (control) 6.13 ± 0.55 mmol/dL (treated)	<0.05
Arain et al., 2025	Rabbit	STZ	36	100 mg/kg b.w.	42 d	583.3 ± 3.58 mg/dL (control at d 42) 201 ± 3.31 mg/dL (treated at d 42)	<0.05

**Table 8:** Meta-analytic results for the lipid profile in CM-treated patients. Data from Khalid et al. [91].

Studies Involved	Total Patients	Parameter	Estimated Mean Difference [95% CI]	*p*-Value
10	322	Total cholesterol (%)	−21.69 [−41.05, −2.33]	0.03
10	322	Triglycerides (%)	−18.79 [−36.16, −3.42]	0.02
7	218	Low-density lipoprotein (%)	−11.92 [−20.57, −3.26]	0.007
7	218	High-density lipoprotein (%)	10.37 [1.90, 18.84]	0.02

## Data Availability

The original contributions presented in this study are included in the article.

## Abbreviations

The following abbreviations are used in this manuscript:

CM	Camel milk
DM	Diabetes Mellitus
T1DM	Type 1 Diabetes Mellitus
T2DM	Type 2 Diabetes Mellitus
